# Microstructures Evolution and Micromechanics Features of Ni-Cr-Si Coatings Deposited on Copper by Laser Cladding

**DOI:** 10.3390/ma11060875

**Published:** 2018-05-23

**Authors:** Peilei Zhang, Mingchuan Li, Zhishui Yu

**Affiliations:** 1 School of Materials Engineering, Shanghai University of Engineering Science, Shanghai 201620, China; 15317538065@163.com (M.L.); yu_zhishui@163.com (Z.Y.); 2 Shanghai Collaborative Innovation Center of Laser Advanced Manufacturing, Shanghai University of Engineering Science, Shanghai 201620, China

**Keywords:** laser cladding, rapid solidification, microstructure, coatings

## Abstract

Three Ni-Cr-Si coatings were synthesized on the surface of copper by laser cladding. The microstructures of the coatings were characterized by optical microscopy (OM), X-ray diffraction (XRD), and scanning electron microscopy (SEM) with an energy dispersive spectrometer (EDS). According to the analysis results of phase compositions, Gibbs free energy change and microstructures, the phases of three coatings appeared were Cr_3_Si+γ-Ni+Cu_ss_ (Coating 1, Ni-26Cr-29Si), Cr_6_Ni_16_Si_7_+Ni_2_Si+Cu_ss_ (Coating 2, Ni-10Cr-30Si) and Cr_3_Ni_5_Si_2_+Cr_2_Ni_3_+Cu_ss_ (Coating 3, Ni-29Cr-16Si). The crystal growth in the solidification process was analyzed with a modified model, which is a combination of Kurz-Giovanola-Trivedi (KGT) and Lipton-Kurz-Trivedi (LKT) models. The dendrite tip undercooling in Coating 2 was higher than those of Coating 1 and Coating 3. Well-developed dendrites were found in Coating 2. A modification of Hunt’s model was adopted to describe the morphological differences in the three coatings. The results show that Coating 1 was in the equiaxed dendrite region, while Coatings 2 and 3 were in the columnar dendrite region. The average friction coefficients of the three coatings were 0.45, 0.5 and 0.4, respectively. Obvious plastic deformation could be found in the subsurface zone of Coatings 2 and 3.

## 1. Introduction

The high thermal and electrical conductivity of copper and its alloys makes them interesting materials for applications in the metallurgical and electrical industries, e.g., in crystallizer used for continuous casting and rolling, commutators of electric motors and blast furnace tuyere [1,2]. However, low strength and poor slide wear resistance shorten the service life of copper alloys’ parts. Among the numerous surface modification methods, laser cladding (LC) has the advantages of metallurgical bonding and mild thermal damage to the substrate, small heat affected zones, low dilution, etc. The wear resistance of Cu can be improved without losing other performances excessively by applying LC to the surface of copper and its alloys [3].

Ni-based coating was synthesized on pure copper by laser cladding using coaxial powder feeding. The microhardness of the coating was HV_0.1_ 360 and the wear resistance was enhanced obviously [4]. Co-based alloy/TiC/CaF_2_ self-lubricating composite coatings were successfully prepared on a Cr-Zr-Cu alloy for continuous casting mold by Yan et al. [5]. The average hardness of the Co-based alloy/20% TiC/10% CaF_2_ (vol.) was 814.3 HV_0.1_; its friction coefficient was reduced to about 0.2 when rubbed with GCr15 tool steel.

It is known that high reflectivity to infrared wavelength and poor wettability with many other materials makes laser cladding on copper substrate difficult [6]. In recent years, many researchers have tried to introduce an intermediate layer to shrink the mismatch in thermal properties, enhance the adhesive strength between the coating and substrate, and reduce the formation of cracks. Triballoy cladding of copper substrates is accomplished by a CW-CO_2_ laser coating with alloys such as NiCrSi in order to improve the energy coupling between the laser and substrate [7]. Ni–Co duplex coating (Ni was prepared as a transition coating) was successfully clad on copper substrate by Liu et al. [8]. The high microhardness and crack free advantages of Ni–Co duplex coating are appropriate to reduce the plastic deformation and adhesive wear of copper substrate. The volume loss of the copper substrate was about seven times the Ni–Co duplex coating. To enhance the wear resistance and, hence, increase the service life of electrical contacts made of Cu, Mo/Ni/Cu “sandwich” layer was fabricated on copper by laser cladding. Ni was introduced as an intermediate layer between Mo and Cu. As a result, the abrasive wear resistance of the clad layer was improved. The specific electrical contact resistance of the clad surface was negligible compared to pure copper [9].

However, the above-mentioned coating may lose efficacy rapidly when copper alloys are used under severe environment. The transition metal silicide Cr_3_Si, with a cubic Al5 crystal structure, has drawn researchers’ attention with respect to applying it as a coating material, due to its outstanding balance of low density, high melting point, unique chemical composition, covalent-dominated strong atomic bonds, excellent elevated-temperature creep strength and high-temperature oxidation resistance [10,11]. Cr_3_Si-reinforced laser clad intermetallic coatings were fabricated on a substrate of austenitic stainless steel 1Cr18Ni9Ti by Wang et al. [12]. The results show that the higher the volume fraction of Cr_3_Si, the higher the hardness and the ambient/high temperature wear and corrosion resistance of the laser clad composite coatings [13].

Cr_6_Ni_16_Si_7_ and Cr_3_Ni_5_Si_2_ have Mg_6_Cu_16_Si_7_-type and AlAu_4_-type structures, respectively [14]. Ternary metal silicides retain the most key features, such as high hardness and high wear resistance, exhibited by most Cr–Si binary metal silicides. They provide the opportunity to fully utilize a large thermodynamically stable composition range, flexible changes of alloying compositions, and intelligent selections of phase-equilibrium [15,16]. They are regarded as promising reinforcements which can be applied to surface modification on copper under a severe environment. Indeed, ternary metal silicides are a large family. Then, Ni-*M*-Si (*M* = Mo, Nb, Cr, Mn, Ti, W) alloy composite materials have been studied widely as important wear-resistant materials. In Ni-Mo-Si alloy composites, Mo_2_Ni_3_Si demonstrates high hardness, excellent ultra-high resistance, high temperature stability, high-temperature oxidation resistance, high-temperature wear resistance, and corrosion resistance [17,18,19,20,21,22,23,24,25,26].

In our previous work, microstructure evolution, mechanical properties, and strengthening mechanisms of Cu-Mo-Si [3], Ni-Ti-Si [27], Ni-Mn-Si [28], and Ni-W-Si [29] systems by laser cladding have been meticulous researched. A (Ti, W)C-reinforced Ni–30Cu metal matrix composite (MMC) coating was deposited on copper by LC. Before depositing the MMC layer, a Ni–30Cu alloy laser-clad interlayer was conducted to overcome the incompatibility between the MMC layer and the copper substrate. An average hardness value of about 811.8HV_0.1_ of the MMC layer was obtained, almost nine times that of the copper substrate. The (Ti, W)C-reinforced MMC coating exhibited higher wear resistance and a lower friction coefficient than those of copper substrate [30].

**Figure 1:**
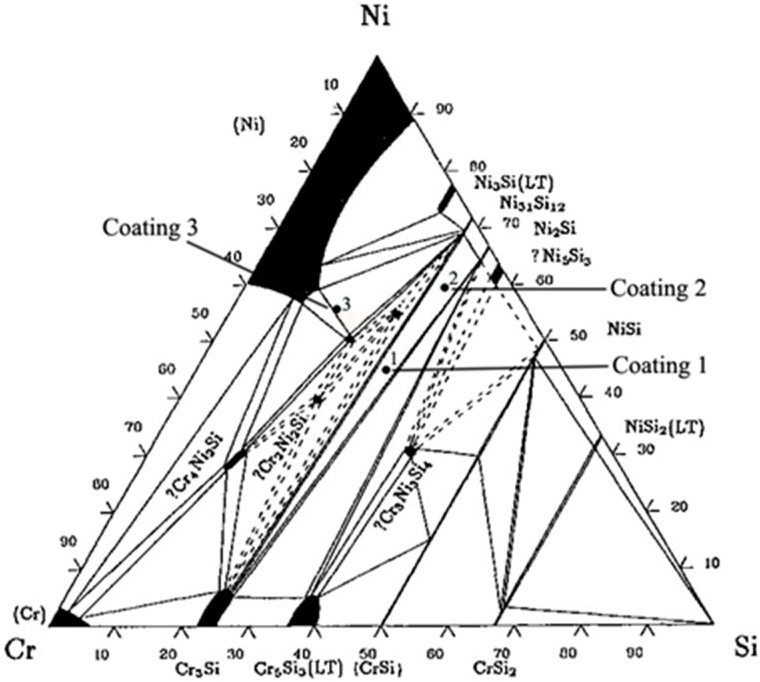
Isothermal section of the Ni-Cr-Si ternary alloy at 1123 K [32].

Ni and Cu have a similar crystal structure and unlimited intersolubility [31]. Some great challenges are whether the Ni-based silicide coatings can be synthesized on copper directly and whether the wear resistance of the materials can be enhanced greatly. In this paper, three Ni-Cr-Si coatings were synthesized on copper by laser cladding. Three coatings were designated as Coating NCRS1, Coating NCRS2 and Coating NCRS3 (hereafter called Coating 1, Coating 2 and Coating 3). To take full advantage of the intersolubility between Ni and Cu, composition points locate in the Ni-rich side of the Ni-Cr-Si ternary alloy phase diagram at 1123 K [32], as shown in Figure 1. Microstructure evolution, Mechanical properties and strengthening mechanisms of three coatings were meticulously researched comparatively. Additionally, comparative studies on microstructure morphological evolution with different compositions of Ni-Cr-Si coatings were conducted by a combination of the KGT and LKT models.

## 2. Materials and Methods

**Figure 2:**
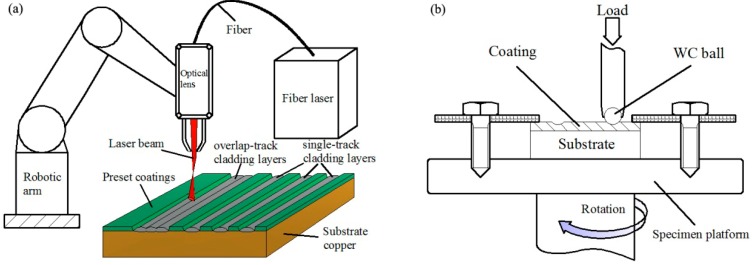
The schematic diagram of (**a**) the fiber laser material processing system (**b**) the wear test system.

In this study, with an average particle size ranging from 50 to 75 μm, Ni-Cr-Si powders (≥99.5% purity, Sinopharm Chemical Reagent Co., Ltd., Shanghai, China) were selected as the precursor materials. The compositions of Ni-Cr-Si powders are shown in Table 1, corresponding to point 1, point 2, and point 3 in Figure 1. The powders (Ni-Cr-Si) were mixed by a ball mill for 30 min and then dried for half an hour at 150 °C before laser processing. The substrates of the test coupon (50 mm × 50 mm × 8 mm) used for LC were pure copper. The copper samples were pre-treated by dilute H_2_SO_4_-HCl solution and sandblasting treatment. The mixed powders were pre-placed onto the substrate surface with a binder (5% cellulose acetate + diacetone alcohol solution). Laser cladding was conducted in the argon shielded chamber of a 5 kW fiber laser material processing system (IPG YLS-5000), and its schematic diagram is shown in Figure 2a. The parameters of laser cladding are shown in Table 1. For each specimen, single-track laser cladding was conducted for microstructure characterization purpose, and six successive overlap tracks clad side-by-side with an overlap ratio of 30% were carried out for wear testing purposes.

Transverse metallographic specimen of the laser-clad Ni-Cr-Si coatings were prepared using standard mechanical polishing and were etched by a solution of 1 mL HF, 3 mL HNO_3_, and 5 mL H_2_O. The microstructure of the coatings was characterized using a VHX-600 optical microscope (OM) and Hitachi S-3400N scanning electron microscope (SEM). Rigaku X-ray diffractometry (XRD-D/max-RB) was used to classify the coatings with Cu Kα irradiation (λ = 0.154060 nm); the scanning speed was 4°/min, and the step size was 0.02°. Chemical compositions of the phase constituents were analyzed by EDS equipped on a Hitachi S-3400N SEM. The average hardness of the coatings were measured using a HXD-1000 automatic Vickers micro-hardness tester with a test load of 100 g (average hardness of alloy) and a load-dwell time of 15 s.

The wear properties of the coatings were evaluated on a CETR-UMT Multi-Specimen Test System at room temperature with an applied load of 49 N, as shown in Figure 2b. The WC (HRA92) ball was used as the contact-coupling ball in the wear test. The diameter of the WC ball and the rotation were 9.5 mm and 4 mm, respectively. The relative sliding speed between the specimen and the contact-coupling ball was 21.4 mm/s. The wear test cycle lasted for 60 min, and the total wear sliding distance was approximately 77 m. This test was meant to investigate the effect of the different composition of contents (Ni-Cr-Si) on the room-temperature wear behaviors of the coatings. So, in order to avoid interference from surface roughness, wear tests were executed on approximately the same surface roughness. In addition, an excessively rough surface interfered with the results of the tribotests. Hence, the surfaces of the coatings were ground successively with 150P, 800P, 1200P, and 2000P SiC abrasive papers in an M-2 pregrinder. Afterward, the samples were cleaned with alcohol in an ultrasonic cleaner and air dried.

The wear volume loss rate was measured by using a VHX-5000 optical microscope (OM) with three-dimensional (3D) depth of field synthesis technology. The friction coefficient was calculated from the friction torque curves recorded with a graphic recorder during the whole sliding wear process.

## 3. Results

### 3.1. Phase Identification and Gibbs Free Energy Calculation

**Figure 3:**
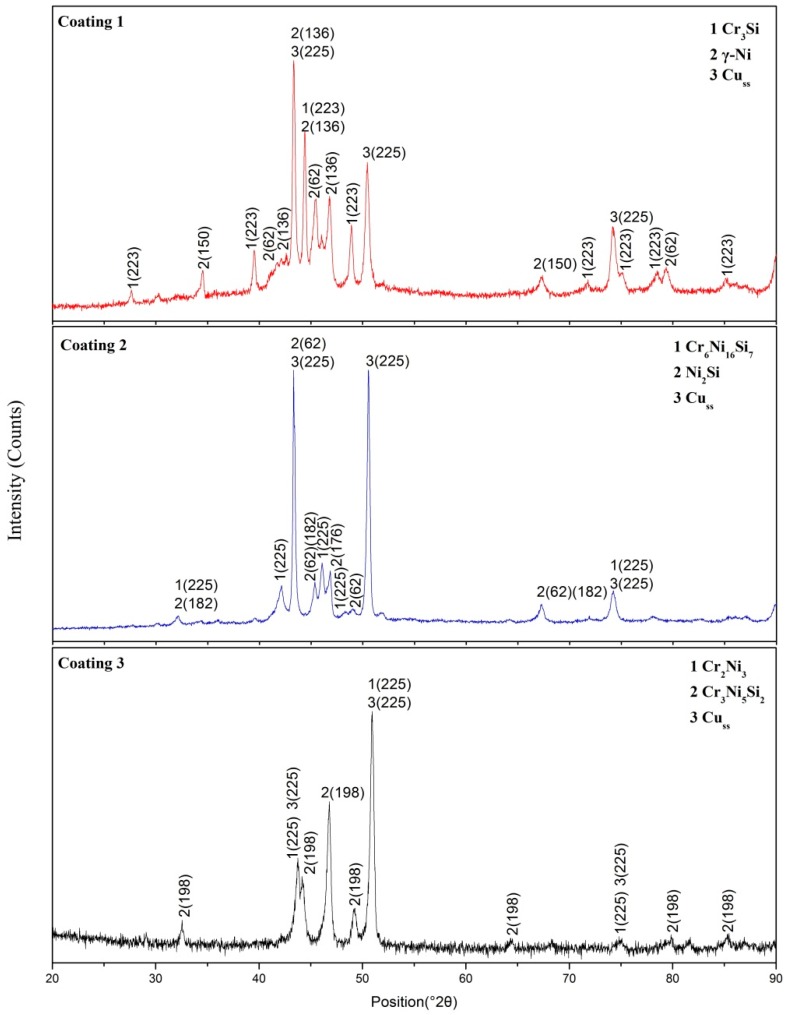
X-ray diffraction (XRD) patterns of the laser cladding coatings with Coating 1, Coating 2, and Coating 3.

The XRD patterns of the laser cladding coatings are shown in Figure 3. It can be seen that the three Ni/Cr/Si atomic ratios of the elemental powders’ blends produced coatings with different phase constitution. Cu-based solid solution (Cu_ss_) was identified in all coatings due to the dilution of the substrate. Owing to the rapid non-equilibrium solidification, the phases generated in the coatings did not abide strictly by the equilibrium phase diagram (Figure 1).

To further evaluate the stability of the phases, the Gibbs free energy change (Δ*G*) of five possible phases was calculated. The mixing Gibbs free energy was calculated using the following relationship [33]:

Δ*G* = Δ*H_mix_* − *T*Δ*S_mix_*, (1)

where Δ*H_mix_* is the mixing enthalpy and approximately equates to $4\sum_{i=1,i\neq j}^{n}c_{i}c_{j}\Delta H_{ij}^{mix}$; $c_{i}$ and $c_{j}$ are the mole percentages of components *i* and *j* of the alloy; $\Delta H_{ij}^{mix}$ is the mixing enthalpy between component *i* and *j*: its values calculated using Miedema’s model are listed in Table 2. *T* is the temperature. Taking into account the effect of atomic size mismatch, Δ*S_mix_* equates to $-R\sum_{i=1}^{n}c_{i}\ln\varphi_{i}$. *R* is the gas constant, 8.314 J/(mol K). $\varphi_{i}[=(c_{i}r_{i}^{3})/\sum_{i=1}^{n}c_{i}r_{i}^{3}]$ is the atomic volume fraction of the *i*th component, and $r_{i}$ is the atomic radius [33].

**Figure 4:**
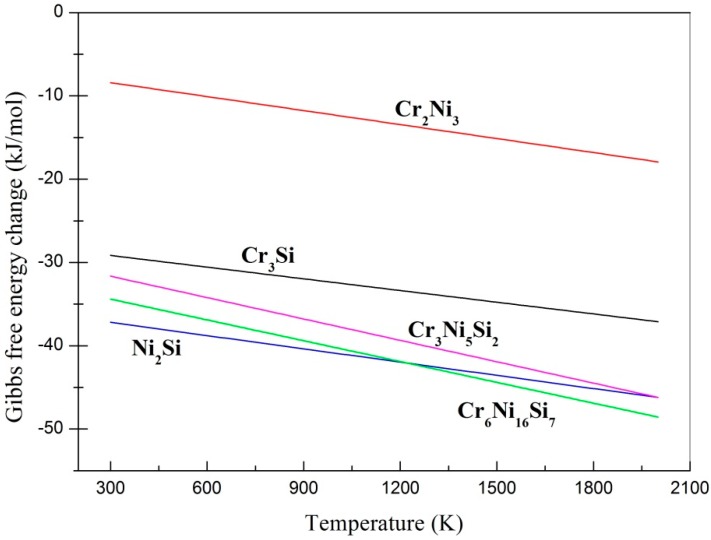
The Gibbs free energy of the synthesis reactions verse temperature.

The relationship between the Gibbs free energy change and temperature is presented in Figure 4. It was found that all five values were below zero, which means that the synthesis reactions could happen. Figure 4 shows that with increasing temperature, the Gibbs free energy decreased. This indicates that the synthesis reaction has a higher possibility of happening under high temperature. It was found that Ni_2_Si had a lower free energy than Cr_3_Si. This does not mean that Ni_2_Si is the most likely phase to be formed in the coatings. For example, Cr_3_Si and γ-Ni were found in Coating 1 but not Ni_2_Si. Cr_3_Si (1770 °C) has a higher melting point than Ni_2_Si (<1455 °C). Therefore, Cr_3_Si phase began to crystallize and separate out before the formation of Ni_2_Si. Ni was enriched in interdendritic Cr_3_Si. At high temperatures, Cr_6_Ni_16_Si_7_ and Cr_3_Ni_5_Si_2_ had lower free energy than Ni_2_Si and Cr_2_Ni_3_, respectively.

### 3.2. Microstructure Analysis

**Figure 5:**
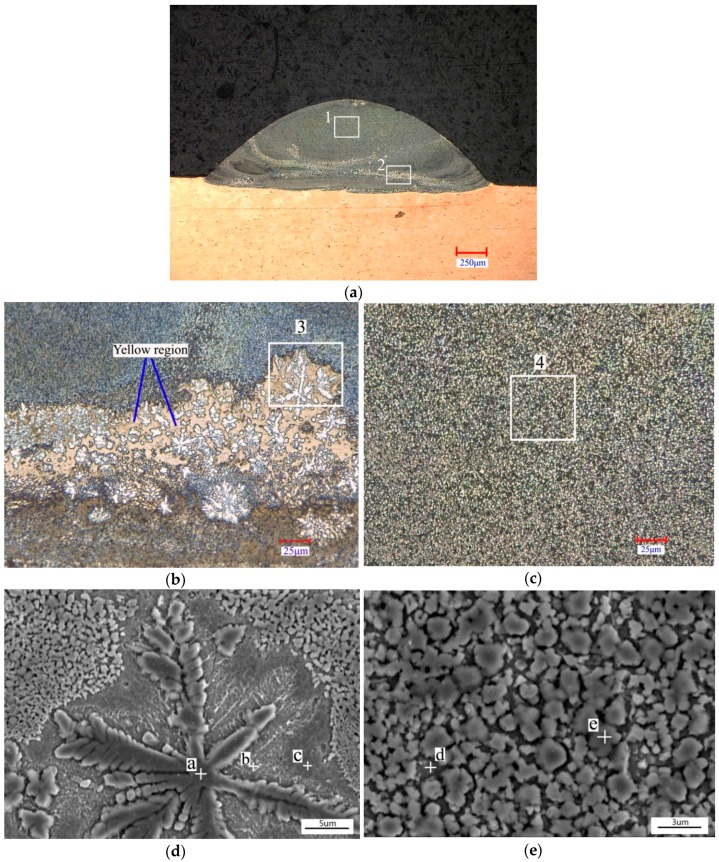
Cross-sectional optical microscopy (OM) images and scanning electron microscopy (SEM) images of Coating 1: (**a**) OM image of Coating 1; (**b**) Amplification of region 2 in image (**a**); (**c**) Amplification of region 1 in image (**a**); (**d**) Amplification of region 3 in image (**b**); (**e**) Amplification of region 4 in image (**c**).

Figure 5 shows the microstructure of Coating 1. The original laser cladding coating was metallurgically bonded to the substrate, and it consisted of two typical microstructures. The- major microstructure of Coating 1 was fine equiaxed grains. Some big equiaxed dendrites appeared at the bottom of the coating corresponding to the “white ribbon” as shown in the overview transverse-section OM photograph of Figure 5a. Figure 5b,c display the amplification of regions 2 and 1 in Figure 5a, respectively. It can be seen from the optical microscope (Figure 5b) that the “white ribbon” is composed of a large number of white big equiaxed dendrites and yellow interdendritic phases. A further amplification of this area is shown in Figure 5d. EDS analysis of points a, b and c (Table 3) was carried out. It indicates that the big equiaxed dendrite was Cr_3_Si. The interdendritic phase was Cu_ss_, and the transitional phase of γ-Ni was found between Cr_3_Si and Cu_ss_. Figure 5e displays the amplification of a typical microstructure. EDS tests of point d and e indicate that the fine equiaxed grain was Cr_3_Si, and the intergranular phase was γ-Ni.

**Figure 6:**
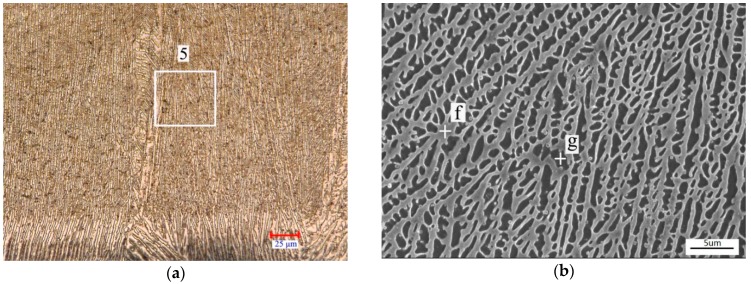
Typical microstructure morphologies of Coating 2: (**a**) OM image of Coating 2; (**b**) Amplification of region 5 in image (**a**).

The typical microstructure of Coating 2 is shown in Figure 6. It is mainly composed of well-developed dendrites at the upper and middle parts of the coating, and columnar grains at the bottom of the coating (Figure 6a). The growth of the dendrites presents obvious directionality. The average diameter of the dendrites was about 1 μm (Figure 6b). According to the EDS analysis of points f and g (Table 3) and Gibbs free energy change (Figure 4), the primary dendrites and columnar grains were Cr_6_Ni_16_Si_7_ and the interdendritic phases were Ni_2_Si.

**Figure 7:**
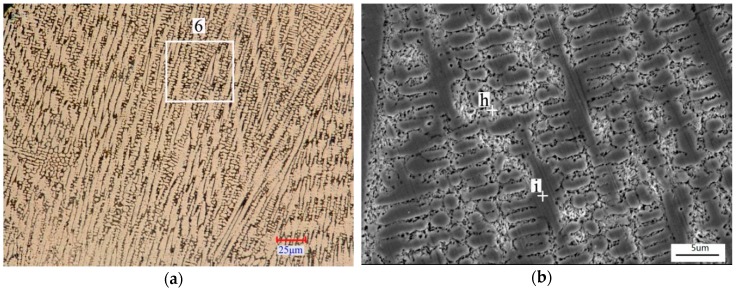
Typical microstructure morphologies of Coating 3: (**a**) OM image of Coating 3; (**b**) Amplification of region 6 in image (**a**).

The average size of the dendrites in Coating 3 was bigger than that of Coating 2. The average diameter of the dendrites in Coating 3 was about 5 μm. The directivity of the dendrites was obvious, as shown in Figure 7a. Interdendritic eutectic structure can be observed in Figure 7b. EDS analysis of points h and i (Table 3) indicates that the well-developed primary dendrites were Cr_3_Ni_5_Si_2_ and that interdendritic eutectics were Cr_2_Ni_3_/Cr_3_Ni_5_Si_2_.

**Figure 8:**
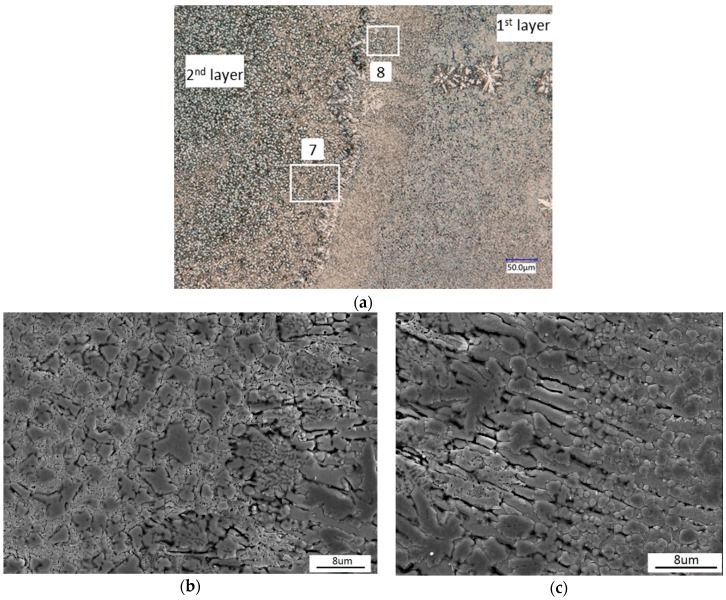
Cross-sectional OM image and SEM image of the overlap region of Coating 1: (**a**) Amplification of region 7 in image (**a**); (**b**) Amplification of region 6 in image (**a**); (**c**) Amplification of region 8 in image (**a**).

The cross-sectional morphologies of the overlap region of Coating 1 are shown in Figure 8. The small area of heat influence is favorable to prevent the grown grains and phase transformations of the previously treated area. Furthermore, some regions of the previously treated area were used as the matrix materials of the next layer and have the same composition and crystal structure. Thus, the similar microstructure in the upper track can be found in the repeat scan. An obvious interface, which was about 25 µm wide, was found between the clad layer and the remelt zone, as can be seen in Figure 8a. Figure 8b,c display the amplification of regions 7 and 8 in Figure 8a, respectively. As clearly shown in Figure 8b,c, the dimensions of the primary dendrites (Cr_3_Si) of the second layer were bigger than those of the first layer. This may be attributed to the effect of heat accumulation by laser scanning twice. It can be seen from Figure 8c that the growth direction of the columnar was nearly perpendicular to the interface of the melted interface. Partial remelting of the substrate (1st layer) directionally solidified under high temperature gradient in an epitaxial growth way.

### 3.3. Analysis of the Solidification Process

Laser cladding has the significant feature of rapid solidification at the molten pool, i.e., non-equilibrium solidification. Thus, the dendritic growth into undercooled alloy melts is extended to the case of large undercooling. The modified model, which is a combination of KGT and LKT models, is presently the best in accordance with the solidification process of laser cladding. The modified model was effectively used in analyzing and understanding the solidification process of Ni-Cr-Si alloy.

The crystal growth in the solidification process was further analyzed using a modification model, which is a combination of Kurz-Giovanola-Trivedi (KGT) [35] and Lipton-Kurz-Trivedi (LKT) models [36]. As laser cladding is analogous to directional solidification, the effective partition coefficient during solidification can be calculated using the following equation [37]:

(2) $$k_{vi}=\frac{a_{0}V/D_{i}+k_{ei}}{a_{0}V/D_{i}+1},$$

where *V* is solidification front velocity (hereinafter referred to as SFV); *k_ei_* is the equilibrium partition coefficient of component *i*; *a*_0_ is the characteristic length of the order of an atomic distance.

With increasing SFV, the extent of solute trapping increases significantly [38]. As a consequence, the above-mentioned phases (XRD results) are supersaturated solid solutions which contain a large number of non-phase composition elements. This can be certified by the EDS results shown in Table 3. For rapid solidification, according to the model, the total undercooling is expressed as a sum of different contributions [39,40]:

(3) $$\Delta T=\Delta T_{t}+\Delta T_{c}+\Delta T_{r}+\Delta T_{k},$$

where Δ*T_t_* represents the thermal undercooling, Δ*T_c_* the solutal undercooling, Δ*T_r_* the curvature undercooling, and Δ*T_k_* the kinetic undercooling.

For solidification of metallic alloys, the thermal contribution (Δ*T_t_*) is much smaller than other undercooling [41]. The Δ*T* of the dendrite tip can be expressed as follows [34,42]:

(4) $$\Delta T=\sum_{i=1}^{n-1}(m_{i}c_{0i}-m_{vi}c_{i}^{*})+\frac{2\Gamma }{R}+\frac{V}{\mu_{k}},$$

where Γ is the Gibbs-Thomson coefficient; *μ_k_* is the linear kinetic coefficient; *m_i_* is the liquidus slope of the component i; *c*_0*i*_ is the nominal concentration of the component *i*; *m_vi_* is the velocity dependent liquidus slope of the component *i*; $c_{i}^{*}$ is the composition of the liquid at the dendrite tip. *R* is the dendrite tip radius: it can be calculated through the application of the following equation:

(5) $$R=2\pi {\left[\frac{\Gamma }{\sum_{i=1}^{n=1}m_{vi}G_{ci}\xi_{ci}-G}\right]}^{\frac{1}{2}},$$

(6) $$m_{vi}=m_{i}\{1+\frac{k_{ei}-k_{vi}\left[1-\ln\left(k_{vi}/k_{ei}\right)\right]}{1-k_{ei}}\},$$

(7) $$G_{ci}=-\frac{Vc_{i}^{*}\left(1-k_{vi}\right)}{D_{i}},$$

(8) $$\xi_{ci}=1-\frac{2k_{vi}}{{\left[1+1/\left(\frac{Pe_{i}^{2}}{4\pi^{2}}\right)\right]}^{\frac{1}{2}}-1+2k_{vi}},$$

(9) $$c_{i}^{*}=\frac{c_{0i}}{1-\left(1-k_{vi}\right)Iv\left(Pe_{i}\right)},$$

where *G_ci_* is the concentration gradient of component *i* in the liquid at the dendrite tip; *ξ_ci_* is the stability parameter; *Pe_i_* is the solute Peclet number for component *i*; *G* is the mean temperature gradient at the interface; *Iv*(*Pe_i_*) can be simplified as follows: *Iv*(*Pe_i_*) = 2*Pe*/(2*Pe* + 1) [39,40].

**Figure 9:**
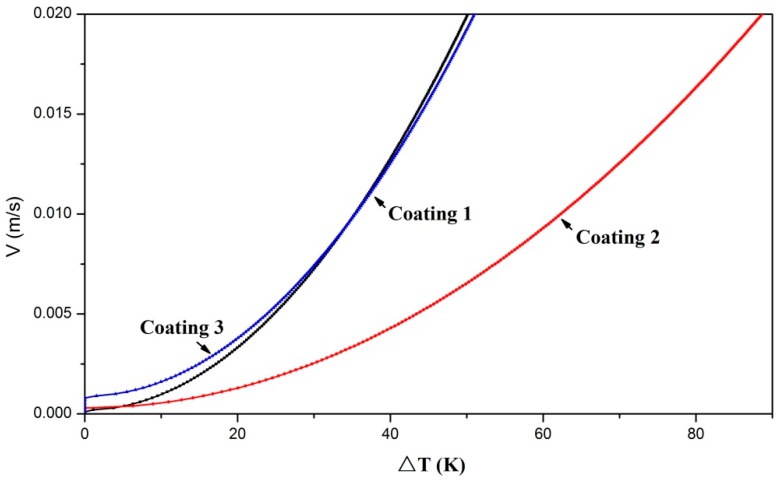
Solidification front velocity with the increase of dendrite tip undercooling (Δ*T*).

SFV with the increase of dendrite tip undercooling Δ*T* are displayed in Figure 9. Δ*T* presents the parabolic growth along with the increase of SFV. Under the laser surface treatment conditions, the thermal gradient *G* was set as 1.5 × 10^6^ K/m [41]. The uniform scanning velocities of laser beam (0.013 m/s) were employed to clad the three coatings; therefore, the solidification velocities were analogous and approximated as 0.01 m/s [43]. It can be seen from Figure 9, under the same SFV, that the dendrite tip undercooling in Coating 2 is higher than that in Coating 1 and Coating 3. Well-developed dendrites were found in Coating 2. Compared to Coating 2, only a few big equiaxed dendrites were found at the bottom of Coating 1. A large number of cellular grains were found in Coating 3. Because of the relative low dendrite tip undercooling, deficient growth impetuses can contribute to the growth of grains in Coating 1 and Coating 3.

This paper focuses on the study of the solidification aspects, and three coatings that were mainly composed of equiaxed grains and dendrites, or columnar dendrites. Hunt’s model was used as analysis of columnar-to-equiaxed grain transition. However, Hunt’s model was too simple to precisely analyze the problem of the non-equilibrium solidification. Then, a modification of Hunt’s model proposed by M. Gaumann [41] was adopted to describe the morphological differences in the three coatings:

(10) $$G=\frac{\Delta T}{n+1}{\left[\frac{-4\pi N_{0}}{3\ln\left(1-\varphi \right)}\right]}^{\frac{1}{3}},$$

where *n* is an alloy parameter; *N*_0_ is the number of nucleation sites; *ϕ* is the volume fraction of nucleation sites (Table 4).

**Figure 10:**
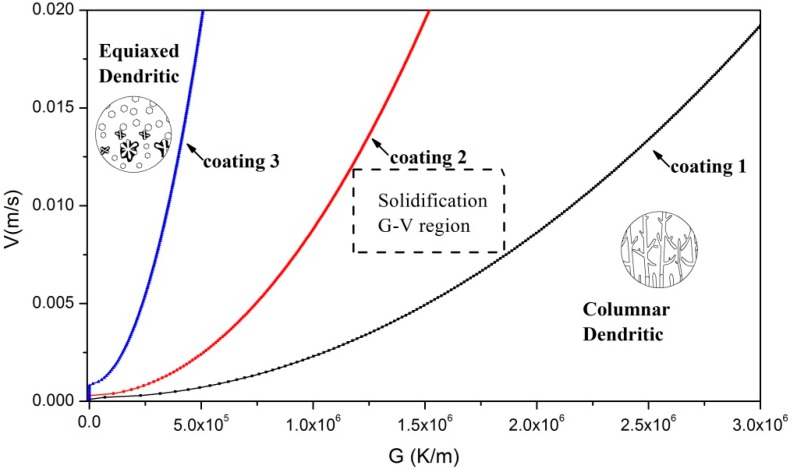
Plot of solidification front velocity *V* against Temperature gradient *G* for Ni-Cr-Si alloys, *N*_0_ = 2 × 10^15^, *ϕ*1 = 0.9, *ϕ*2 = 0.05, *ϕ*3 = 0.01.

Hunt proposed that fully equiaxed growth occurred if the volume fraction of equiaxed grains *ϕ* > 0.49, whereas the structure was assumed to be fully columnar if *ϕ* < 0.0066 [44,45]. It is shown in Figure 5a that the volume fraction of globular equiaxed grains in Coating 1 was more than 90 percent; hence, *ϕ* is 0.9. As shown in Figure 6b, a few fine equiaxed grains appeared in the interdendritic region of Coating 2; so, *ϕ* is 0.05. Scarcely any equiaxed grains appeared in Coating 3; hence, *ϕ* is 0.01. The microstructure selection map is presented in Figure 10. Under 0.01 m/s and 1.5 × 10^6^ K/m [41], Coating 1 was at the left-hand side of the limit-line (equiaxed dendrite region). Therefore, the microstructure was composed of primary fine equiaxed grains and a few big equiaxed dendrites. Columnar dendritics and cellular grains could not be found in Coatings 1, 2 and 3 which were located at the right-hand side (columnar dendrite region) of their limit line, respectively, and the representative microstructure was composed of columnar dendrites under the same condition.

**Figure 11:**
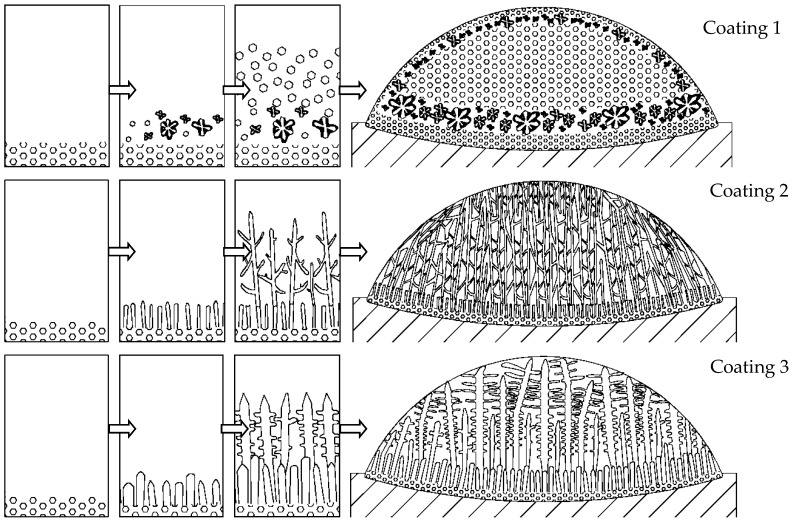
Schematic illustration of microstructures evolution in solidification process of three coatings.

A schematic illustration of microstructure evolution during the solidification process is shown in Figure 11. At the first stage of solidification, chill equiaxed crystal layers were induced by copper matrix at the bottom of the coatings. The solidification G-V region, shown in Figure 10, lies at the left-hand side of the limit-line of Coating 1 and the right-hand side of Coatings 2 and 3. As the solidification continues, equiaxed grains were generated in Coating 1, while cellular trunks were generated in Coatings 2 and 3. After further growth, some equiaxed grains in Coating 1 turned into big equiaxed dendrites, cellular trunks in Coating 2 grew into well-developed dendrites, and cellular trunks in Coating 3 grew into columnar dendrites. During the grain growth process, Δ*T* in Coating 2 was large (Figure 9). Tertiary dendrite arm may be generated; eventually, well-developed or “net-like” dendrites were found in Coating 2. Δ*T* in Coatings 1 and 3 were low. There were not enough growth impetuses for grains in Coatings 1 and 2 to grow into well-developed crystals. As a result, only a few big equiaxed dendrites were found in Coating 1; instead, Coating 1 was mainly composed of fine equiaxed grains. Under-developed columnar dendrites, which contained some secondary dendrites on the primary dendrites arms, were found in Coating 3.

### 3.4. Micromechanics Features

**Figure 12:**
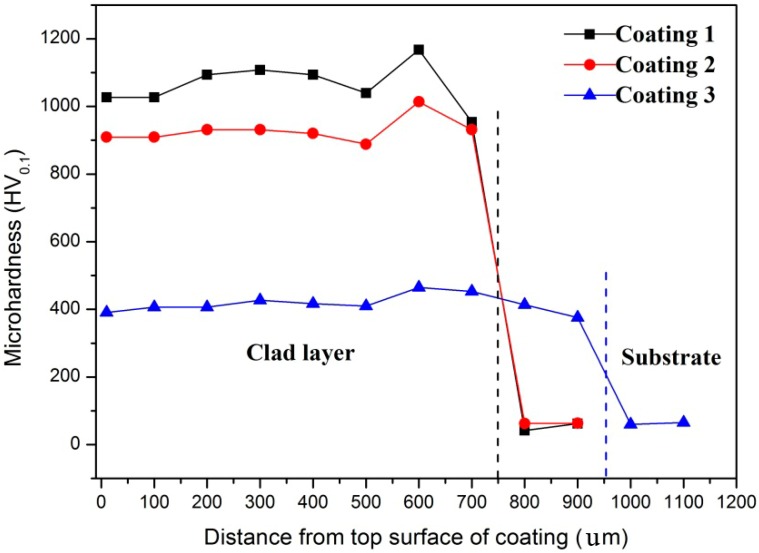
Variations of microhardness along the depth of coatings.

Variations of microhardness along the depth of the three coatings with different Ni/Cr/Si contents are shown in Figure 12. It can be found that the thickness of the coating was 750, 750, and 950 µm for Coating 1, 2 and 3, respectively. Every value of hardness shown in Figure 12 is an average value of the three points measured. It is seen that the average microhardness of Coatings 1, 2 and 3 were about 1050 HV_0.1_, 900 HV_0.1_, and 400 HV_0.1_, respectively.

### 3.5. Tribological Behavior

**Figure 13:**
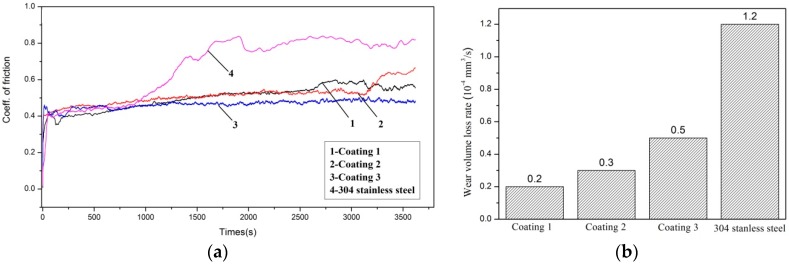
(**a**) Friction coefficient and (**b**) wear volume loss rates of laser cladding Ni-Cr-Si coatings and the reference materials 304 stainless steel.

**Figure 14:**
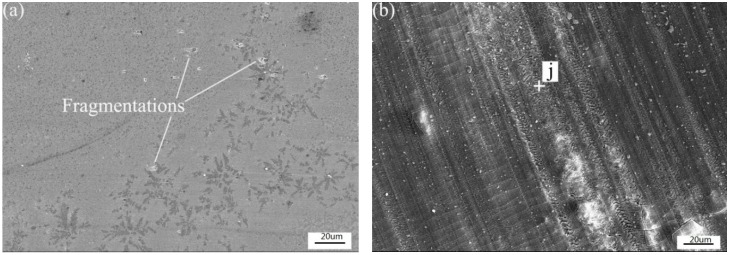

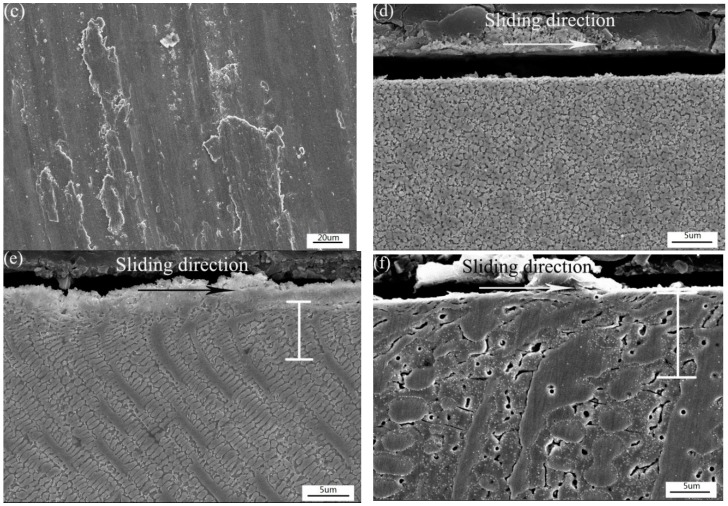
SEM micrographs showing the worn surfaces and sections perpendicular morphologies of Coating 1 (**a**,**d**), Coating 2 (**b**,**e**) and Coating 3 (**c**,**f**).

The friction coefficients of the laser cladding coatings under the same conditions are shown in Figure 13a. It can be observed that the average friction coefficient of Coatings 1, 2 and 3 were 0.45, 0.5 and 0.4, respectively due to the microhardness and uniformity of the microstructure. The friction coefficient curves of the laser cladding coatings were relatively smooth. But the curve of the reference material (304 stainless steel, 200 HV_0.1_) showed a high friction coefficient value and large fluctuation. At an early stage, the frictional contact region of Coating 1 was the fine equiaxed grains region (Figure 14d). The friction coefficient curve was smooth and steady. After about 43 min, a variation appeared in the curve. This is as a result of the exposure of big equiaxed dendrites (Figure 14a). The big equiaxed dendrites were Cr_3_Si. The big interdendritic region was γ-Ni and Cu_ss_ was around the big equiaxed dendrites. Cr_3_Si has high hardness in comparison with γ-Ni and Cu_ss_. During friction, the interdendritic phase was more quickly worn off in comparison with the big equiaxed dendrites. The big dendrites were protruded. Hence, the surface roughness of the rubbing surfaces was enhanced. As a result, friction was increased. Then, the friction coefficient curve rises and becomes and wavy. The primary dendrites and columnar grains were Cr_6_Ni_16_Si_7_, and the interdendritic phases were Ni_2_Si in Coating 2. Ni_2_Si, as an interdendritic compound, has strong atomic bonds but low toughness [42]. Ni_2_Si has a high hardness, but it cannot be compared with Cr_3_Si. Cr_6_Ni_16_Si_7_ has a higher hardness and excellent toughness, because of its covalent and metallic bonds. In addition, owing to the drawback of the apparatus for friction-wear tests, some wear debris might go into the wear scar at a later period of the test. The debris would increase the frictional force between the coating and the counterpart and then cause a variation of the friction coefficient curve of Coating 2. So, the Cr_2_Ni_3_ and Cr_3_Ni_5_Si are the main phases of Coating 3. Cr_2_Ni_3,_ an intermetallic compound, and Cr_3_Ni_5_Si, with an AlAu_4_-structure, have high hardness and excellent toughness. As a result, Coating 3 had excellent hardness and toughness. The friction coefficient curve of Coating 3 was smoother and a little lower than other curves despite the fact that the microhardness of Coating 3 was only about 400 HV_0.1_.

Further investigation of wear resistance was carried out. The wear volume loss rates of the three coatings and reference material are shown in Figure 13b. The wear volume loss rates were 0.2 × 10^−4^ mm^3^/s (Coating 1), 0.3 × 10^−4^ mm^3^/s (Coating 2), 0.5 × 10^−4^ mm^3^/s (Coating 3) and 1.2 × 10^−4^ mm^3^/s (reference material), respectively. For all investigations, the laser cladding coatings possessed lower friction coefficient and less wear volume loss rate compared with the 304 stainless steel. The wear volume loss rate did not increase with increasing friction coefficient. The wear volume loss rate of Coating 1 were approximately the same as those of Coating 2 but were considerably lower than those of Coating 3. The bulgy nature of Cr_3_Si prevented its surface from wearing off. Thus, wear volume loss rates were low in Coating 1. The hardness of Coating 2 was higher than that of Coating 3, because of Ni_2_Si and Cr_6_Ni_16_Si_7_. In addition, the debris that covered the surface is an effective way to prevent wear and tear. So, the wear volume loss rate of Coating 2 was lower than those of Coating 3.

As shown in Figure 14a, the wear scar surface of Coating 1 was smooth except for some fragmentations. The excellent abrasion resistance of Coating 1 can be attributed to the covalent-dominated strong atomic bonds of Cr_3_Si. Fragmentations were found in the worn out surfaces of Coating 1. Due to the insufficient sustenance of the hard phase (Cr_3_Si) by γ-Ni in the interdendritic region, some hard phases would peel off from the surface of the coating.

In Figure 14b, the EDS results (Point j: O 53.79-Si 11.94-Ni 21.96-Cr 8.69-Cu 3.62 at %) showed that the wear scar surface layer of Coating 2 was rich in oxygen. It was deduced that the debris went back to the wear scar. As a result, it caused the variation of the friction coefficient. A slightly adhesive wear surface micrograph is presented in Figure 14c. Low frictional force can be applied as a combination of abrasive wear and slight adhesive wear mechanism in Coating 3. There were no noticeable features of plastic deformation or observable micro-cracks in the subsurface zone of the wear scar (Coating 1), as shown in Figure 14d. On the contrary, Coating 2 and Coating 3 suffered plastic deformation during dry sliding wear process, as indicated in Figure 14e,f. There were slight plastic flows along the rolling direction. For Coating 1, because of the high microhardness (HV_0.1_ ≈ 1050), plastic deformation did not easily happen in the wear test. Besides, the microstructure of equiaxed grains and equiaxed dendrites are non-directional. No plastic deformation was found in the subsurface of the wear scar. The thickness of the deformation layer in Coating 2 was less than that in Coating 3 (Figure 14e,f) because of the different hardness.

Based on the results of the above analyses, it is found that the three coatings with different Ni-Cr-Si compositions (Table 1) have different wear volume loss rates and friction coefficients. With increase of hardness of the coatings, the wear volume loss rates were reduced. However, the friction coefficients were irrelevant to the hardness of the coatings. The friction coefficient depends on composition, surface roughness and so on. The friction coefficient is a comprehensive property. In addition, lower friction coefficient does not mean better wear resistance. The wear resistance is a comprehensive property in main relation to the hardness and the other mechanical parameters of materials.

## 4. Conclusions

Three Ni-Cr-Si coatings were synthesized on the surface of copper using laser cladding. According to the analysis results of phase composition, Gibbs free energy change and microstructure, the phases of three coatings were Cr_3_Si+γ-Ni+Cu_ss_ (Coating 1), Cr_6_Ni_16_Si_7_+Ni_2_Si+ Cu_ss_ (Coating 2) and Cr_3_Ni_5_Si_2_+Cr_2_Ni_3_+Cu_ss_ (Coating 3).

The crystal growth in the solidification process was analyzed using a modification model, which is a combination of KGT and LKT model. A higher dendrite tip undercooling was found in Coating 2 than those in Coatings 1 and 3. Therefore, well-developed dendrites were found in Coating 2. A modification of Hunt’s model was adopted to describe the morphological differences among Coatings 1, 2 and 3. The results show that Coating 1 was located in the equiaxed dendrite region. However, Coatings 2 and 3 were located in the columnar dendrite region. This conforms to the microstructure characteristics of the three coatings.

The average microhardness of Coatings 1, 2 and 3 were about 1050 HV_0.1_, 900 HV_0.1_, and 400HV_0.1_ respectively. Their average friction coefficients were 0.45, 0.5, and 0.4, respectively. In Coating 1, there were no noticeable features of plastic deformation in the subsurface of the wear scar. Obvious plastic deformation in the subsurface zone can be found in Coatings 2 and 3. A combination of abrasive wear and slight adhesive wear can be described as the wear mechanism.

## Figures and Tables

**Table 1:** Compositions of Ni-Cr-Si powders and laser parameters.

Coating Number	Powder Composition			Laser Parameters		
	Ni (at %)	Cr (at %)	Si (at %)	Power (W)	Scan Speed (mm/min)	Diameter of Laser Beam (mm)
1	45	26	29	4500	800	3.5
2	60	10	30	4500	800	3.5
3	55	29	16	4500	800	3.5

**Table 2:** Values of $\Delta H_{ij}^{mix}$ (kJ/mol) calculated for atomic pairs between elements [34] and atomic radius.

Mixing Enthalpy	Ni	Cr	Si	Atomic Radius/pm
Ni	-	−7	−40	135
Cr	-	-	−37	140
Si	-	-	-	110

**Table 3:** Energy dispersive spectrometer (EDS) analyses of test points in coatings.

Region	Element Composition (at %)			
	Ni	Cr	Si	Cu
a	3.11	69.32	27.57	—
b	41.18	10.57	26.69	21.56
c	17.77	2.86	8.65	70.73
d	6.92	61.96	26.43	4.69
e	33.8	25.79	22.15	18.25
f	52.49	18.03	20.17	9.3
g	56.29	7.13	27.25	9.33
h	46.24	26.23	18.51	9.02
i	47.82	25.73	9.42	17.03

**Table 4:** Physical parameters of Ni-Cr-Si alloy used for the calculation of solidification process.

Parameter	Value	Ref.
Liquidus temperature of Coating 1, T_m1_	2040 K	Obtained using CALPHAD
Liquidus temperature of Coating 2, T_m2_	1650 K	Obtained using CALPHAD
Liquidus temperature of Coating 3, T_m3_	1580 K	Obtained using CALPHAD
Slope of liquidus surface with respect to chromium concentration, m_Cr1_, m_Cr2_, m_Cr3_	−3.63, −3.63, 5.87 K/(at %)	Obtained using CALPHAD
Slope of liquidus surface with respect to nickel concentration, m_Ni1_, m_Ni2_, m_Ni3_	−10.34, −12.62, −10.72 K/(at %)	Obtained using CALPHAD
Equilibrium partition coefficient for chromium, k_Cr1_, k_Cr2_, k_Cr3_	0.264, 0.264, 0.2044	Obtained using CALPHAD
Equilibrium partition coefficient for nickel, k_Ni1_, k_Ni2_, k_Ni3_	0.243, 0.295, 0.305	Obtained using CALPHAD
Pre-exponential diffusion coefficient for chromium, D_Cr0_	2.67 × 10^−7^ m^2^/s	[40]
Pre-exponential diffusion coefficient for nickel, D_Ni0_	4.92 × 10^−7^ m^2^/s	[40]
Activation energy for diffusion for chromium, Q_Cr_	6.69 × 10^4^ J/mole	[40]
Activation energy for diffusion for nickel, Q_ni_	6.77 × 10^4^ J/mole	[40]
Length scale for solute trapping, a_0_	5 × 10^−9^ m	[40]
Gibbs–Thomson coefficient, Γ	2.47 × 10^−7^ Km	[41]
Linear kinetic coefficient, μ_k_	4.696 m/s K	[41]
Alloy parameter, n	3.4	[43]
The number of nucleation sites, N_0_	2 × 10^15^/m^3^	[43]
